# Evolution of Increased Photosynthetic Capacity and Its Underlying Traits in Invasive *Jacobaea vulgaris*

**DOI:** 10.3389/fpls.2019.01016

**Published:** 2019-08-08

**Authors:** Tiantian Lin, Peter G. L. Klinkhamer, Thijs L. Pons, Patrick P. J. Mulder, Klaas Vrieling

**Affiliations:** ^1^College of Forestry, Sichuan Agricultural University, Chengdu, China; ^2^Institute of Biology, Plant Ecology and Phytochemistry, Leiden University, Leiden, Netherlands; ^3^Plant Ecophysiology, Institute of Environmental Biology, Utrecht University, Utrecht, Netherlands; ^4^RIKILT – Wageningen University & Research, Wageningen, Netherlands

**Keywords:** evolution of increased competitive ability hypothesis, shifting defense hypothesis, invasion ecology, photosynthetic capacity, pyrrolizidine alkaloids, root carbohydrate storage

## Abstract

The evolution of increased competitive ability (EICA) hypothesis and the shifting defense hypothesis (SDH) predict that evolutionary changes occur in a suite of traits related to defense and growth in invasive plant species as result of the absence of specialist herbivores. We tested how this suite of traits changed due to the absence of specialist herbivores in multiple invasive regions that differ in climatic conditions with native and invasive *Jacobaea vulgaris* in a controlled environment. We hypothesized that invasive *J. vulgaris* in all invasive regions have i) a higher plant growth and underlying traits, such as photosynthetic capacity, ii) lower regrowth-related traits, such as carbohydrate storage, and iii) an increased plant qualitative defense, such as pyrrolizidine alkaloids (PAs). Our results show that invasive *J. vulgaris* genotypes have evolved a higher photosynthetic rate and total PA concentration but a lower investment in root carbohydrates, which supports the SDH hypothesis. All the traits changed consistently and significantly in the same direction in all four invasive regions, indicative of a parallel evolution. Climatic and soil variables did differ between ranges but explained only a very small part of the variation in trait values. The latter suggests that climate and soil changes were not the main selective forces on these traits.

## Introduction

Invasive plants provide an excellent opportunity for ecologists to study evolutionary changes by considering invasions as large-scale and long-term experiments in which major alterations in selective forces have occurred (Turner et al., 2014). One of the most frequently studied selective forces is the release from specialist natural enemies. Since defenses against herbivores are costly (Koricheva, 2002; Velzen and Etienne, 2015) and many defensive traits are genetically controlled (Fritz and Simms, 1992; Kariñho‐Betancourt et al., 2015), a shift in the herbivore composition towards a guild that is dominated by generalist herbivores is expected to exert an altered selective pressure on invasive plants. The evolution of increased competitive ability (EICA) hypothesis (Blossey and Nötzold, 1995) and the shifting defense hypothesis (SDH) (Müller-Schärer et al., 2004; Joshi and Vrieling, 2005) argue that a release from specialist herbivores leads to a decreased investment in costly quantitative defenses and an increased investment in less costly qualitative defenses. The net decrease in the costs of antiherbivore strategies allows for an increased investment in growth (Colautti et al., 2004; Doorduin and Vrieling, 2011). As a result, invasive plants might outcompete local plant species. The release of specialist herbivores, therefore, leads to specific evolutionary changes in the allocation pattern to growth, defense, and regrowth after defoliation.

Higher growth rates are expected to be accompanied by a change in underlying factors such as increased photosynthesis, higher specific leaf area (SLA, the ratio of total leaf area and leaf dry mass), and higher leaf mass fraction (LMF, the ratio of leaf dry mass and total dry mass) (Poorter and Remkes, 1990; Poorter, 1999; Shipley, 2006). In addition, photosynthetic nitrogen use efficiency (PNUE), the rate of photosynthetic capacity per unit nitrogen, has been reported to be positively associated with specific leaf area and plant growth when plants grow under the same conditions (Poorter and Evans, 1998). Indeed, several invasive plant species have an increased growth compared to their native genotypes, and some have been observed to have higher photosynthetic capacity (*A*_sat_), SLA, LMF, and PNUE (Durand and Goldstein, 2001; Feng et al., 2009; Feng et al., 2011; Qing et al., 2012).

Common ragwort (*Jacobaea vulgaris*) is native to Eurasia and was introduced into parts of Australia (Harper and Wood, 1957), New Zealand (Poole and Cairns, 1940), the east coast of North America since the 1850s, and the west coast of North America since 1900 (Harris et al., 1971). In its native range, monocarpic *J. vulgaris* is under strong selection pressure by several specialist herbivores with the cinnabar moth (*Tyria jacobaeae*) and the fleabeetle (*Longitarsus jacobaeae*) being the most prominent. *Tyria jacobaeae* regularly completely defoliates flowering plants in its native range (Van Der Meijden et al., 2000). Although in the native range *J. vulgaris* is under strong selection of specialist herbivores, it is also expanding here and behaving as a colonizer. In the invasive range, the herbivore guilds of *J. vulgaris* have been reported to be mainly dominated by local generalist herbivores (Poole and Cairns, 1940; Stastny et al., 2005).

Although evolutionary changes in growth rates and antiherbivore defenses are predicted by the EICA and SDH and have been observed in several invasive species (Jakobs et al., 2004; Feng et al., 2009; Van Kleunen et al., 2010b; Uesugi and Kessler, 2016; Zhang et al., 2018), there is no compelling evidence that the absence of specialist herbivores is the main responsible selective force as other biotic or abiotic factors cannot be ruled out as being important for driving selection (Liu and Stiling, 2006; Van Kleunen et al., 2010a; Felker-Quinn et al., 2013; Colomer‐Ventura et al., 2015; Stastny and Sargent, 2017). In addition, several studies have put forward that different locations in the invasive range may provide different results (Moloney et al., 2009; Williams et al., 2010). The latter may result from different herbivore pressures in the invasive areas or from climatic differences resulting in varying conditions of abiotic stress. Ecological niche modeling showed that, for many species, climatic variables can very well explain the distribution areas of the species in the invasive range. For a set of 50 studied species, 15% had more than 10% of their invaded distribution outside their native climatic niche (Petitpierre et al., 2012). However, other traits like soil condition and disturbance can also influence plant traits like growth, defenses, and tolerance (Mckechnie and Sargent, 2013; Yelenik et al., 2017). Therefore, in this study, we compared changes in four invasive regions taking climatic and soil factors into account.

In previous studies, we have compared plant growth, structural defense, root–shoot ratio, and competitive ability of invasive and native genotypes of *J. vulgaris* and found evidence for evolutionary changes in pyrrolizidine alkaloid concentration and composition, regrowth ability after herbivory, and inulin allocation to roots associated with the absence of specialist herbivores (Lin et al., 2015a; Lin et al., 2015b; Lin et al., 2018). Here, we aimed to corroborate our previous results with a more comprehensive data set and to disentangle the underlying features that resulted in increased growth rates in invasive areas such as SLA, LMF, and PNEU.

We compared invasive and native *J. vulgaris* genotypes and hypothesized that invasive *J. vulgaris* in all invasive regions 1) have a higher growth rate, *A*_sat_, SLA, LMF, and PNUE, 2) have a lower root carbohydrate storage capacity, and 3) have higher levels of qualitative defense (pyrrolizidine alkaloids). Finally, we expected that 4) the studied traits change in the same direction due to the lack of specialist herbivores. All these traits were studied in the introduced genotypes from four geographically and climatically distinct regions (Australia, New Zealand, and West and East coast of North America).

## Material and Methods

### Study Species

*Jacobaea vulgaris* (synonym *Senecio jacobaea*) is a monocarpic, perennial, plant that belongs to the Asteraceae family. Ragwort populations of the west and east coast of North America are geographically isolated and thought to be independent introductions. The amount of neutral genetic variation (AFLPs) of native *J. vulgaris* populations does not differ from that of the different invasive regions, suggesting that introductions from multiple source populations have occurred (Doorduin et al., 2010). An assignment analysis using AFLP and chloroplast DNA indicated that populations from the northwest coast of Europe including the UK are the most likely source populations (Doorduin et al., 2010; Doorduin et al., 2011).

*Jacobaea vulgaris* is recorded to contain more than 37 different pyrrolizidine alkaloids (PAs), which function as constitutive, qualitative defenses against herbivores (Witte et al., 1992; Cheng et al., 2011). Approximately 50–100% of the phenotypic variance in the concentration and composition of PAs is under genetic control (Vrieling et al., 1993). Since PAs are toxic to horses and cattle, which can result in significant livestock losses due to PA poisoning and in decreased pasture yields, it received a pest status in the introduced range (Bain, 1991). In a common garden experiment, invasive *J. vulgaris* had, on average, a 90% higher total amount of PAs (especially the Jacobine-like PAs) than genotypes from the native range (Joshi and Vrieling, 2005). PAs play an important role in plant resistance to several generalist herbivores (Van Dam et al., 1995; Macel et al., 2005; Leiss et al., 2009). These specialist herbivores cause the majority of damage in Western Europe, and their distributions overlap with the distribution of ragwort. The cinnabar moth and the flea beetles are adapted to PAs and even sequester PAs for their own use (Aplin and Rothschild, 1972; Zoelen and Van Der Meijden, 1991; Dobler et al., 2000). *Tyria jacobaeae* has been reported to use PAs as oviposition and feeding stimulants (Macel and Vrieling, 2003; Bernays et al., 2004; Cheng et al., 2013). In the past decades, *Tyria jacobaeae* and *L. jacobaeae* were introduced as biological control agents into to all the invasive regions (Syrett, 1983; Bain, 1991; McEvoy et al., 1991; McLaren et al., 2000). But so far, no evolutionary adaptation of invasive *J. vulgaris* populations has been observed after the exposure to introduced *L. jacobaeae* (Joshi and Vrieling, 2005; Rapo et al., 2010).

### Plant Material and Growth Conditions

**Figure 1:**
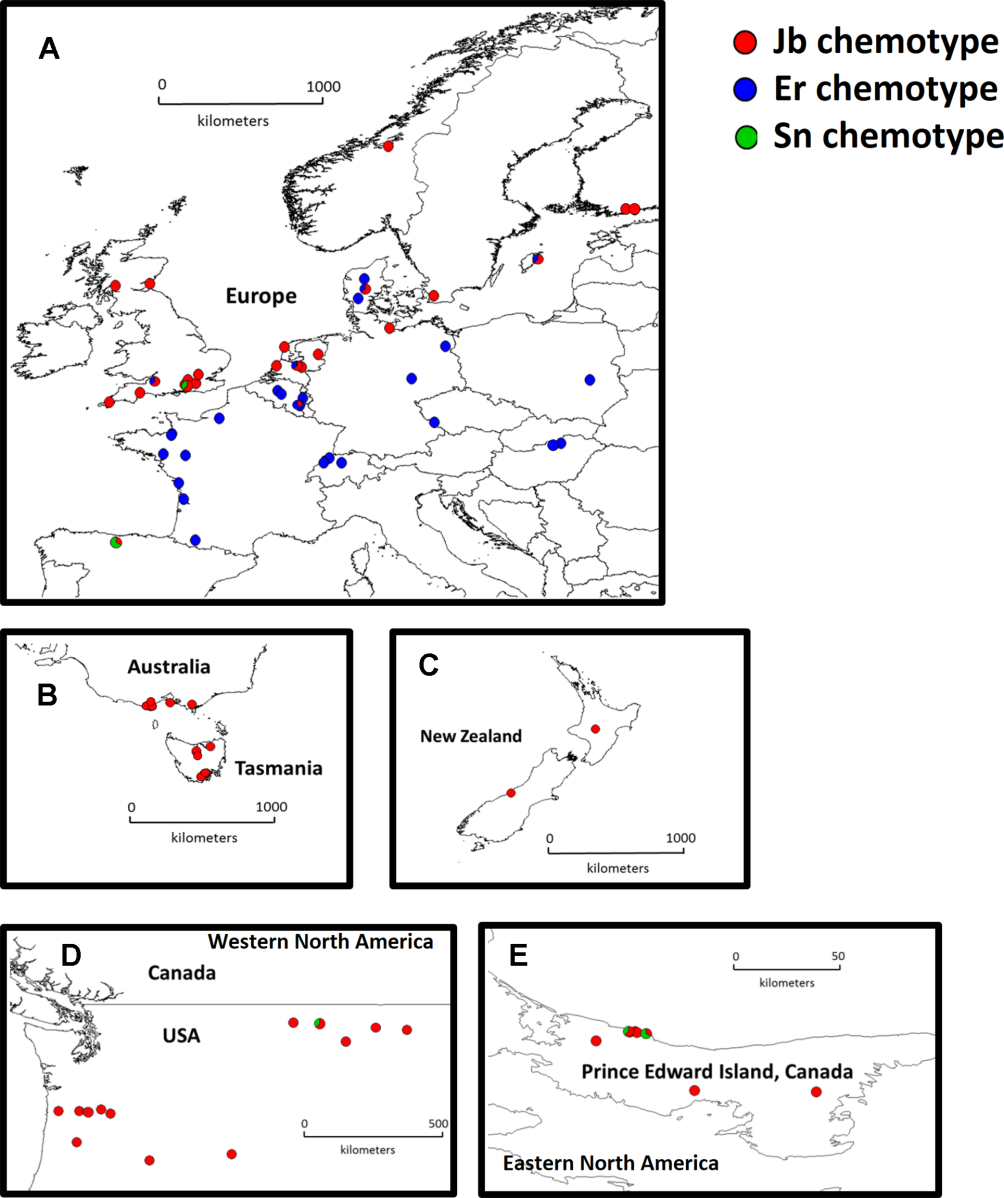
Distribution map of native and invasive *Jacobaea vulgaris*. Europe (**A**, n = 46 populations), Australia (**B**, n = 10 populations), New Zealand (**C**, n = 2 populations), Western North America (**D**, n = 13 populations) and Eastern North America (**E**, n = 6 populations). Different colors in the pie charts indicate different chemotypes in each population and the proportion of different colors in one population indicate the number of genotypes from corresponding chemotypes (1 or 2 out of 3 mother plants).

As we intended to study broad patterns of evolutionary change upon a change in herbivory guild, we sampled a large number of populations across regions at the expense of studying lower number of sampled individuals. Seeds were collected from 46 native populations in Europe and from 31 invasive populations in Australia, New Zealand, Western North America, and Eastern North America (**Figure 1** and see **Table S1** in **Supporting Information**). For each population, seeds from three different (mother) plants were germinated in Petri dishes with moistened filter paper. Two weeks after germination, one well-grown seedling from each mother plant was potted in 0.5-L pots with 20% potting soil (Slingerland potgrond, Zoeterwoude, The Netherlands), 80% sandy soil from the dunes (collected from Meijendel, The Netherlands, 52°13’N, 4°34’E), and 0.75 g Osmocote slow release fertilizer (Scott^®^, Scotts Miracle-Gro, Marysville, OH, USA; N/P/K/MgO 15:9:11:2.5). In a previous study, the fresh weight of 600 seedlings from 20 native and 20 invasive populations were measured at the same age (2 weeks after germination), and no significant differences were found between the two ranges (Lin et al., 2015b). The experiment contained (46 native populations + 31 invasive populations) × 3 (replicates) = 231 plants. Plants were grown in the climate chamber for 9 weeks at 20°C, 70% humidity, 16 h day light with a photosynthetic photon flux density (PPFD) of 113 µmol m^−2^ s^−1^. Eight native plants that showed very poor and stunted growth were excluded from the measurements.

### Photosynthesis and Leaf Nitrogen Measurement

After 6 weeks of growth in the climate chamber, the light saturated rate of photosynthesis at atmospheric CO_2_ concentration per unit leaf area (*A*_sat_) was measured on the middlemost leaf of each plant, using a LICOR 6400 (Portable Photosynthesis analyser, LI-COR Inc. Lincoln, NE, USA) at atmospheric CO_2_ concentration (ca. 380 µmol mol^−1^ in the leaf chamber), at 20°C growth temperature, and a PPFD of 1,500 μmol m^−2^ s^−1^. Before the measurement, leaves were prelighted for 5 min at a high light intensity to initiate photosynthetic induction. To assure that the photosystem is fully saturated, the leaf was prelighted, and the measurement was carried out at a high light intensity. Three measurements were done on the same leaf when steady state was reached, which were averaged and further treated as one data point. The leaf sample used for the photosynthesis measurement was dried in an oven at 60°C for 3 days, and nitrogen (N) concentration was measured with a CHN analyzer (Carlo Erba, Milan, Italy) to calculate the nitrogen content per unit leaf area. In a previous study, the nitrate concentration was analyzed in the leaves of *J. vulgaris*, and only trace amounts of nitrate were found, indicating that *J. vulgaris* is not a nitrate accumulator, and we therefore used total nitrogen content to calculate PNUE.

### Growth Measurements

Until 9 weeks, growth of ragwort plants is exponential (Vrieling and Van Wijk, 1994), and we therefore harvested plants after 9 weeks of growth. All 223 plants were harvested, and the total leaf area of each plant was measured by a portable leaf area meter (LI-3100, LI-COR Inc., Lincoln, NE, USA). Dry mass of shoots and roots were measured separately after oven drying at 60°C for 3 days. Leaf and root materials that were kept apart for later analysis were also accounted in the dry mass and the leaf area. SLA (cm^2^ g^−1^) was calculated as the ratio between total leaf area and shoot dry mass. LMF (g g^−1^) and RMF (g g^−1^) were calculated as the ratio between shoot dry mass or root dry mass and plant total dry mass, respectively. Petioles and stems were negligible in the rosettes of *J. vulgaris* and represented a shoot mass fraction of <1%. PNUE (μmol CO_2_ g^−1^ s^−1^) was calculated as the ratio of *A*_sat_ to leaf N per leaf area.

### Pyrrolizidine Alkaloids Extraction and Analysis

After 9 weeks of growth, two middle leaves of each plant were freeze-dried, and ∼10 mg of powdered leaf material was extracted with 1 ml of 2% formic acid containing 1 µg ml^−1^ heliotrine as an internal PA standard. The plant extract solution was shaken for 30 min. Solid plant material was removed by centrifugation at 720×*g* for 10 min, and the supernatant was filtered through a 0.2-µm nylon membrane (Acrodisc 13-mm syringe filter, Pall Life Sciences, Ann Arbor, MI, USA). An aliquot of the filtered solution (25 µl) was diluted with 10 mM ammonia (975 µl) and 10 µl was injected into the LC-MS/MS system (Acquity UPLC system coupled to a Quattro Premier tandem mass spectrometer, Waters, Milford, MA, USA). PAs were separated on a Waters UPLC BEH C_18_ (150 × 2.1 mm, 1.7 µm) analytical column using a 12-min acetonitrile/water (pH 12) gradient, running from 0 to 50% acetonitrile. The analytical method included 44 PAs, of which 20 were available as reference standards. Analysis by LC-MS/MS was conducted according to Cheng et al. (2011), and the instrumental settings are available as supporting information. Data processing was conducted with Masslynx 4.1 software (Waters Corporation, Milford, MA, USA).

PAs can occur as tertiary amine (free base) and as N-oxides (Areshkina, 1950; Hartmann and Toppel, 1987). According to the structural characteristics and biosynthetic pathway, PAs were classified into four groups: senecionine (Sn)-, jacobine (Jb)-, erucifoline (Er)- and otosenine (Ot)-like PAs (Pelser et al., 2005; Cheng et al., 2011). Based on the relative presence or absence of the Sn-, Jb-, and Er-like PAs, *J. vulgaris* individuals were classified as Sn, Jb, or Er chemotypes (Witte et al., 1992; Macel et al., 2004).We considered plants containing in their PA composition more than 25% of Jb-like PAs and no or little Er-like PAs as Jb chemotypes. Plants containing more than 25% of Er-like PAs and no or little Jb-like PAs were considered as Er chemotypes and plants that had <25% of Er- and Jb-like PAs but larger amounts of Sn-like PAs as Sn chemotypes. Since Ot-like PAs in *J. vulgaris* individuals were present in quantities of 1.3% or less, Ot chemotypes were not identified. Joshi and Vrieling (2005) showed that Er chemotypes are almost absent from the invasive regions. Therefore, we also compared PA concentration and the level of N-oxides between native and invasive Jb chemotypes.

### Root Inulin Analysis

In Asteraceae carbohydrates are stored as inulin (Tertuliano and Figueiredo-Ribeiro, 1993). The root inulin concentration was measured as the difference between the total sugar content after hydrolysis with inulinase and the free sugar content before hydrolysis. Hundred milligrams ground dried root material was extracted with 4 ml distilled water at 80°C for 1 h. After centrifuging, the free sugar content was measured by adding 2 ml 3,5-dinitrosalicylic acid (DNS) to 1 ml of the supernatant and measuring the absorbance with a spectrophotometer at 540 nm. The concentration was calculated using a calibration line made from D-(−)-fructose according to Miller (1959). The total sugar content was determined by hydrolysis of the remaining 1 ml supernatant with 200 µl inulinase (Novozym^®^960, Sigma-Aldrich) for 1 h at 60°C and measurement by the same DNS method. Root total inulin content was calculated as the root inulin concentration multiplied by total root dry mass. In a subset of plants, the inulin content was also measured in the leaves. The inulin concentration in the leaves was <10% of that in the roots and did not differ between native and invasive individuals (data not shown).

Since root biomass consists of both carbohydrate storage and structural tissues, a distinction was made between the carbohydrate storage root dry mass (= root inulin content) and the structural root dry mass (= total root dry mass − root inulin content). The root inulin to structural root ratio (root inulin content divided by structural root mass) and the shoot to structural root ratio were calculated as indicators for regrowth ability and growth ability, respectively.

### Climate and Soil Variables

Since different climates and soil variables in invasive areas could have selected for changed allocation patterns in *J. vulgaris*, we examined the difference in the local climate and soil conditions among the five geographic regions (Europe, Australia, New Zealand, Western North America, and Eastern North America). All 19 available bioclimatic variables of the current conditions (ca. 1950–2000) were downloaded from the WorldClim dataset (http://www.worldclim.org/current) at 5 arc-min resolution for each sampled population (**Table S2**). In addition, 10 available soil variables were downloaded from the world soil information (ISRIC, https://www.isric.org/explore/soilgrids) at 1 km/250 m spatial resolution for each sampled population (**Table S3**).

### Statistical Analysis

To test for traits variation in regions, nested ANOVAs were performed with region (four invasive and one native) as a fixed factor and population nested within region as a random factor. For all traits that differed significantly (with alpha < 0.1) among the five regions *post hoc* LSD tests were carried out. Differences between the native and the invasive range were determined from the results of the *post hoc* test.

Normality of the residuals was checked with a Shapiro–Wilk test. A log transformation was conducted for SLA, PNUE, shoot–structural root ratio, root inulin–structural root ratio, and all PAs. All analyses were carried out using SPSS 18.0 (SPSS: An IBM Company, Wacker Drive, Chicago, USA).

A principal component analysis (PCA) was performed with the SIMCA-P software (v.11.0, Umetrics, Umeå, Sweden) for classifying all sampled populations based on the 19 climatic and 10 soil variables. The scaling method for the PCA was unit variance. Another PCA was performed for classifying all sampled populations from different regions based on 20 growth, regrowth, and defense traits measured in this study (**Table 1**). The scaling method for the PCA was unit variance. The PC scores of the PCA plot of traits related to growth, regrowth, and defense were analyzed using Pearson correlations (**Figure S2**), and the differences in residuals between native and invasive populations were tested using an ANOVA combined with a *post hoc* test.

**Table 1:** Summary of the *p* values of nested ANOVA with region as a fixed factor and population nested within region as a random factor. Dependent variables are growth and underlying growth traits, pyrrolizidine alkaloid (PA) concentrations, inulin content and concentration of *J. vulgaris* of four invasive regions and the native region.

Traits	Variables	p (region)	p (population)
Growth and photosynthesis	Total dry mass (g)	0.059*	0.007
	Leaf mass fraction (g g^−1^)	<0.001	0.013
	Root mass fraction (g g^−1^)	<0.001	0.013
	Specific leaf area (SLA, cm^2^ g^−1^)	0.068*	0.011
	Structural root dry mass (g)	NS	NS
	Shoot- structural root ratio (g g^−1^)	0.04	NS
	Leaf N content per unit area (g m^−2^)	NS	0.043
	*A*_sat_ (μmol CO_2_ m^−2^ s^−1^)	0.025	NS
	PNUE (μmol CO_2_ g^−1^ s^−1^)	0.016	0.001
Root carbohydrate storage	Root inulin concentration (g g^−1^ DM)	<0.001	0.045
	Root total inulin content (g)	0.003	0.001
	Free sugar concentration in root (g g^−1^ DM)	NS	0.001
	Root inulin–structural root ratio (g g^−1^)	<0.001	0.031
Pyrrolizidine alkaloids	Total PA (µg g^−1^ DM)	0.002	<0.001
	Total PA (tertiary amines) (µg g^−1^ DM)	<0.001	<0.001
	Total PA (N-oxides) (µg g^−1^ DM)	NS	<0.001
	Senecionine-like PAs (µg g^−1^ DM)	0.001	<0.001
	Jacobine-like PAs (µg g^−1^ DM)	<0.001	<0.001
	Erucifoline-like PAs (µg g^−1^ DM)	<0.001	<0.001
	Otosenine-like PAs (µg g^−1^ DM)	0.045	NS

p (regions): significance level of nested ANOVA among regions, df = 1, 4; p (populations): significance level of nested ANOVA among populations, df = 1, 72; NS, not significant. Structural root dry mass is the root dry mass minus root inulin content. Root inulin to structural root ratio is calculated as the root inulin content divided by structural root dry mass. *One-sided significant.

## Results

### Growth and Photosynthesis

**Figure 2:**
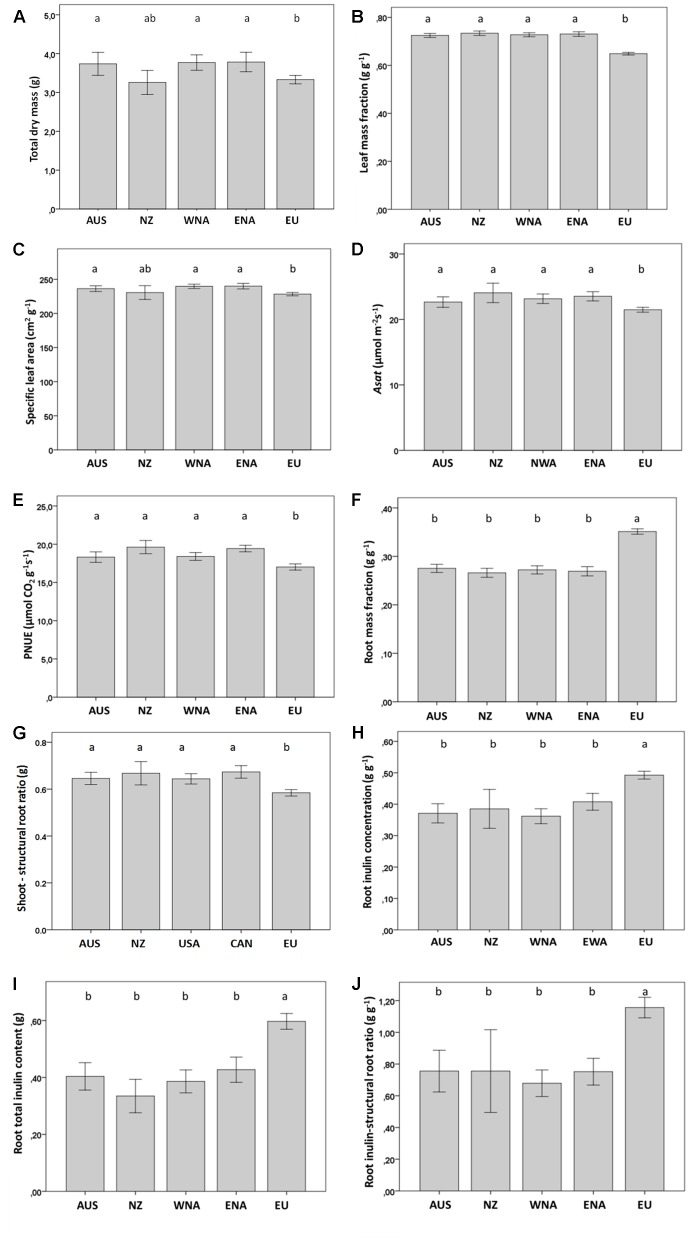
Average value of growth and regrowth related traits from the five regions (Aus, Australia; NZ, New Zealand; WNA, Western North America; ENA, Eastern North America and EU, Europe). **(A)** total dry mass, **(B)** leaf mass fraction, **(C)** specific leaf area, **(D)** Asat, **(E)** PNUE, **(F)** root mass fraction, **(G)** shoot to structural root ratio, **(H)** root inulin concentration, **(I)** root total inulin content, **(J)** root inulin-structural root ratio. Values are means ± SE. Different letters indicate significant differences among regions at p < 0.05 according to a *post hoc* LSD test (for the nested ANOVA results see **Table 1**), without letters there are no differences.

Invasive *J. vulgaris* genotypes from the four invasive regions had, on average, a 12% larger total dry mass (**Table 1**, *p* = 0.059, one-sided significant, **Figure 2A**), a 12% higher LMF (**Figure 2B**), and a 5% larger SLA (**Table 1**, *p* = 0.068, one-sided significant, **Figure 2C**) than native genotypes. Photosynthetic rates of invasive *J. vulgaris* genotypes from the four regions were significantly higher. *A*_sat_ increased by 7.7% and PNUE by 10.8% (**Figures 2D, E**, **Table 1**). Leaf nitrogen content per unit leaf area did not differ between native and invasive genotypes (**Table 1**).

### Root Carbohydrate Storage

*J. vulgaris* genotypes from all four invasive regions had, on average, a 23% smaller RMF than native genotypes (**Figure 2F**). While the structural root mass (total root mass minus inulin mass) showed no difference among the genotypes from the four invasive regions and the native range (**Table 1**), invasive genotypes had, on average, a 15% higher shoot to structural root ratio than native genotypes (**Figure 2G** and **Table 1**). In addition, invasive *J. vulgaris* genotypes from four invasive regions had, on average, a 23 and 34% lower root inulin concentration and total root inulin content, respectively, compared to the native genotypes (**Figures 2H, I**), resulting in a 37% lower root inulin to structural root ratio in invasive genotypes (**Figure 2J**). For the free sugar concentration, no difference was found between the invasive and native range (**Table 1**) although the free sugar content of Eastern North America was lower than that of Australia, Western North America, and Europe.

### Pyrrolizidine Alkaloids

In the native range, two major chemotypes were present. The Jb chemotype (45% of the populations) is predominant in the coastal areas of Northwest Europe (**Figure 1A**, red dots). In contrast, the Er chemotype (54% of the populations) is predominant along the southwest coast and inland Europe (**Figure 1A**, blue dots). Only one population (1%) classified as an Sn chemotype. In the invasive range, 96% of the chemotypes were Jb chemotypes, and the remaining ones were Sn chemotypes (**Figures 1B–E**).

**Figure 3:**
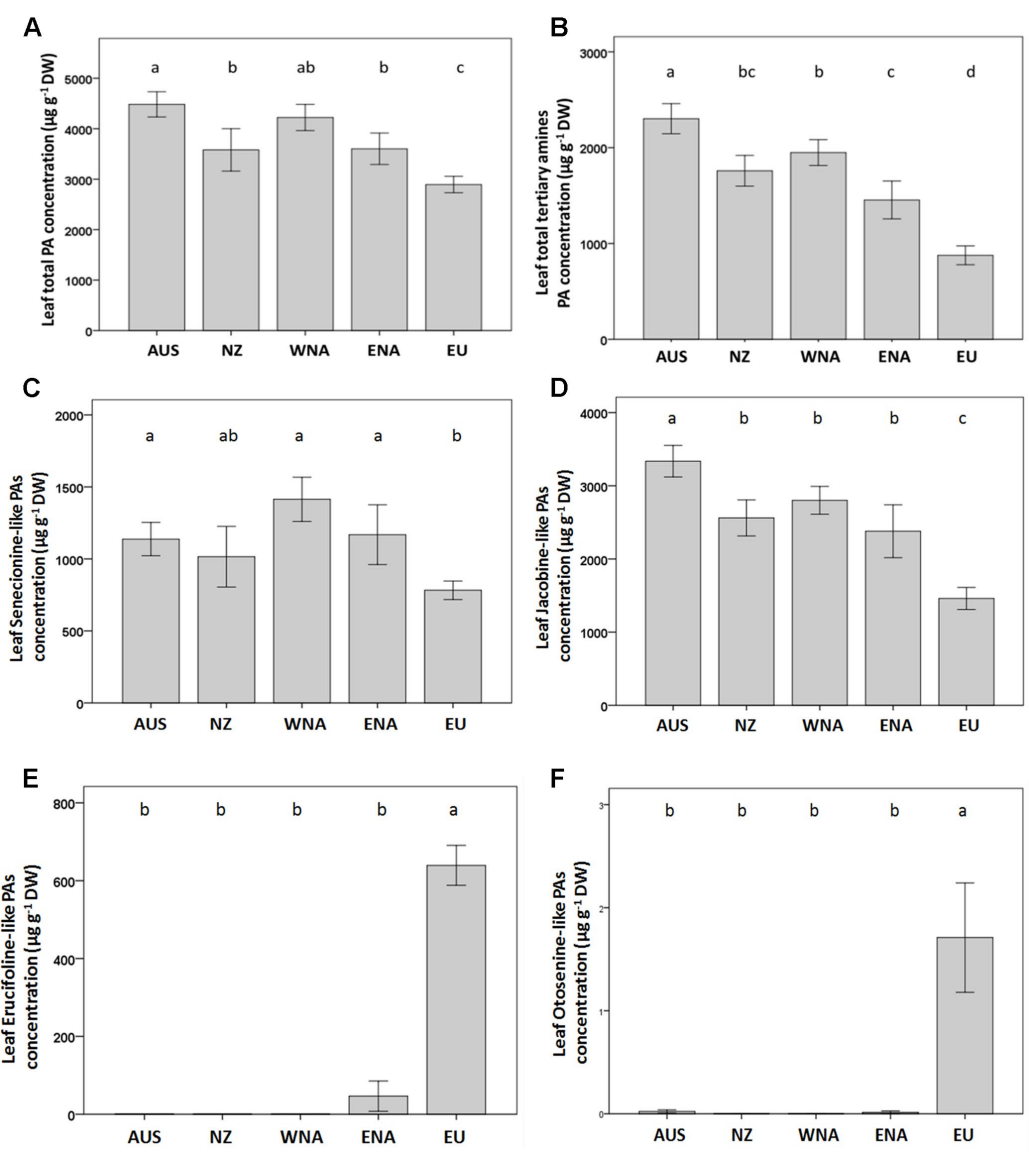
Average value of PA traits from the five regions (Aus, Australia; NZ, New Zealand; WNA, Western North America; ENA, Eastern North America and EU, Europe). **(A)** leaf total PA concentration, **(B)** leaf total tertiary amines PA concentration, **(C)** leaf senecionine-like PA concentration, **(D)** leaf jacobine-like PA concentration, **(E)** leaf erucifoline-like PA concentration, **(F)** leaf otosenin-like PA concentration. Values are means ± SE. Different letters indicate significant differences among regions at p < 0.05 according to a *post hoc* LSD test (for the nested ANOVA results see **Table 1**), without letters there is no differences.

On average, the invasive genotypes from the four regions had a 43% higher total leaf PA concentration than the native genotypes (**Figure 3A**). The PA tertiary amine concentration in the invasive genotypes was 123% higher than that of the native genotypes (**Figure 3B**), while no significant increase was found for the PA N-oxide concentration (**Table 1**). The invasive genotypes contained higher levels of Sn- and Jb-like Pas, while Er- and Ot-like PAs were almost absent in the invasive range (**Figures 3C–F**).

When considering the Jb chemotypes from the four invasive regions and the native region only, no significant differences were found in the levels of total PAs, PA N-oxides, and Jb-like PAs (**Table 2**). However, the invasive Jb chemotypes contained significantly higher PA tertiary amine concentrations than native Jb chemotypes (**Figure S1** and **Table 2**). In addition, the invasive Jb chemotypes contained significantly more Sn-like PAs but less Er- and Ot-like PAs compared to native genotypes (**Figures S1b–S1d** and **Table 2**). This indicates that in invasive Jb chemotypes the PA composition and concentration have changed and that these changes occurred in the same direction for all four invasive regions.

**Table 2:** Summary of the *p* values of nested ANOVA with region as a fixed factor and population nested within region as a random factor. Dependent variables are mean pyrrolizidine alkaloid concentration of Jb chemotypes of *J. vulgaris* from four invasive and the native region.

Variable	p (region)	p (population)
Total PA	NS	0.022
Total PA (Tertiary amines)	0.036	0.001
Total PA (N-oxides)	NS	0.018
Senecionine-like PAs	0.024	<0.001
Jacobine-like PAs	NS	0.005
Erucifoline-like PAs	<0.001	<0.001
Otosenine-like PAs	0.041	0.001

### Principal Component Analysis of Climatic and Soil Variables and Plants Traits

**Figure 4:**
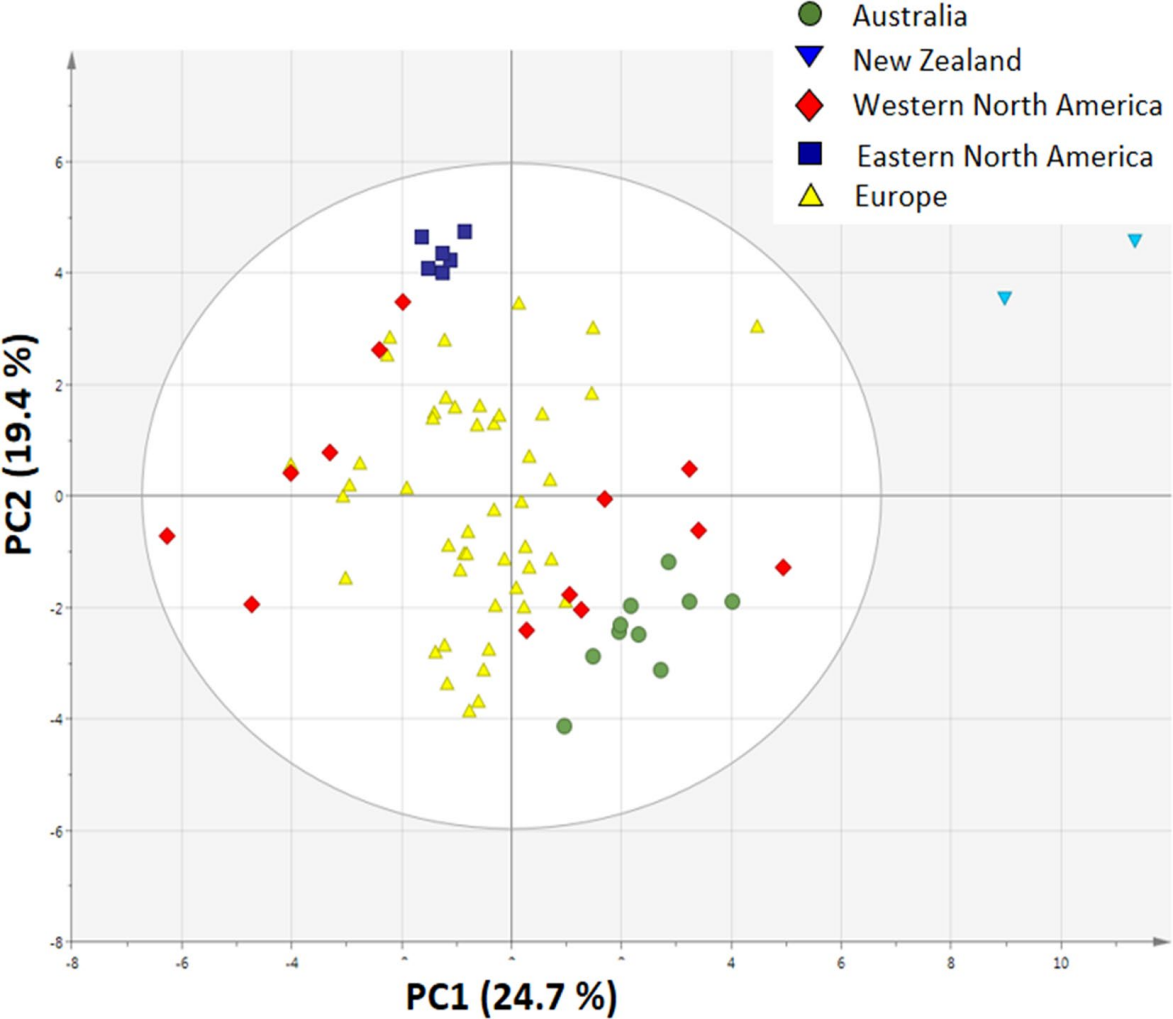
Principal component analysis plot of 19 bioclimatic variables and 10 soil variables from the site of each sampled population (n = 10 for Australia, n = 2 for New Zealand, n = 13 for Western North America, n = 6 for Eastern North America and n = 46 for Europe).

The PCA plot based on 19 climatic and 10 soil variables shows that the invasive populations are all separated from each other and that they are clustered around the native populations, showing that climatic conditions and soil characteristics differed between the invasive regions (**Figure 4**).

**Figure 5:**
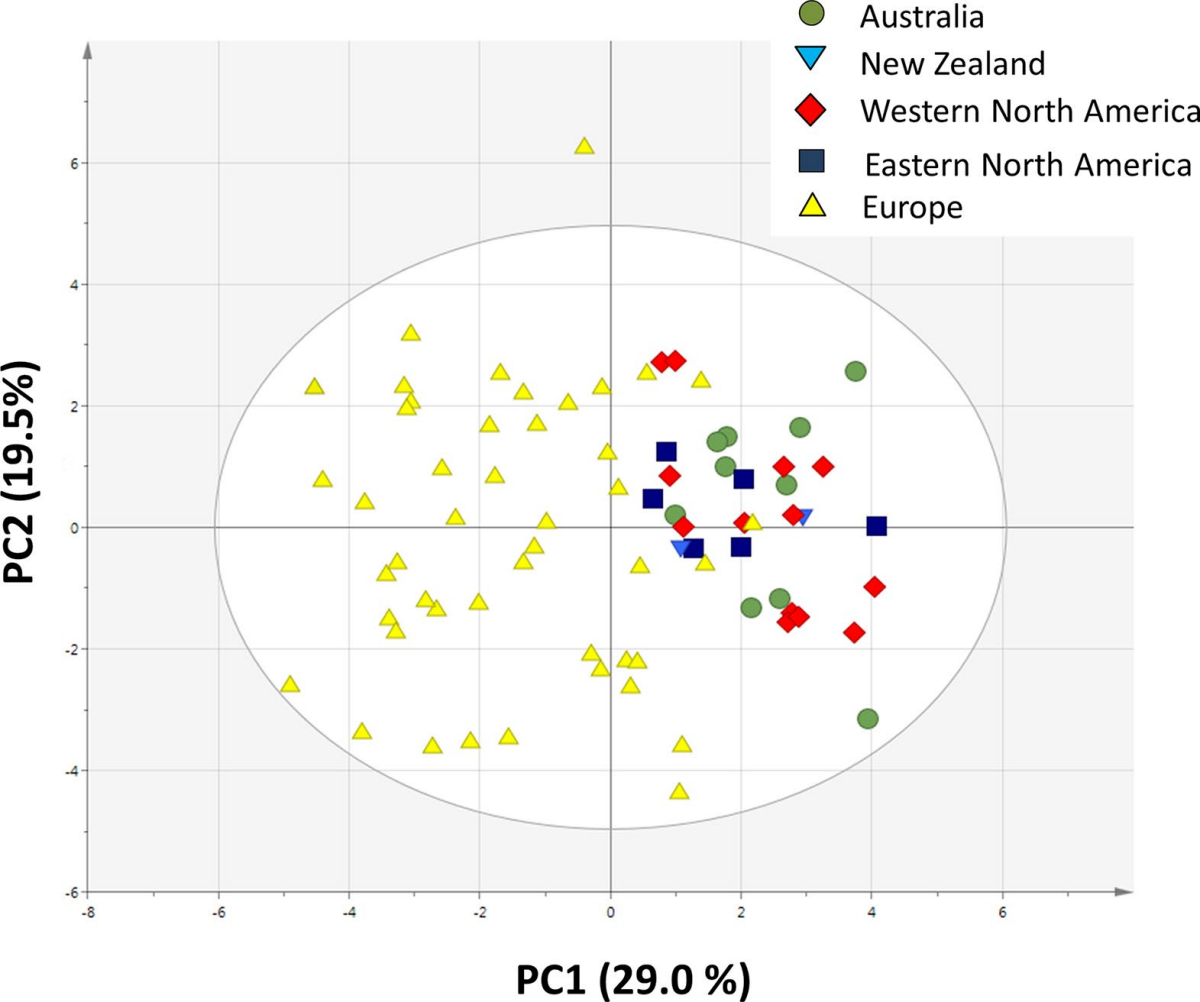
Principal component analysis plot of all 20 traits measured in this study (**Table 1**). For each trait the average value of each population was used (n = 10 for Australia, n = 2 for New Zealand, n = 13 for Western North America, n = 6 for Eastern North America and n = 46 for Europe).

**Figure 6:**
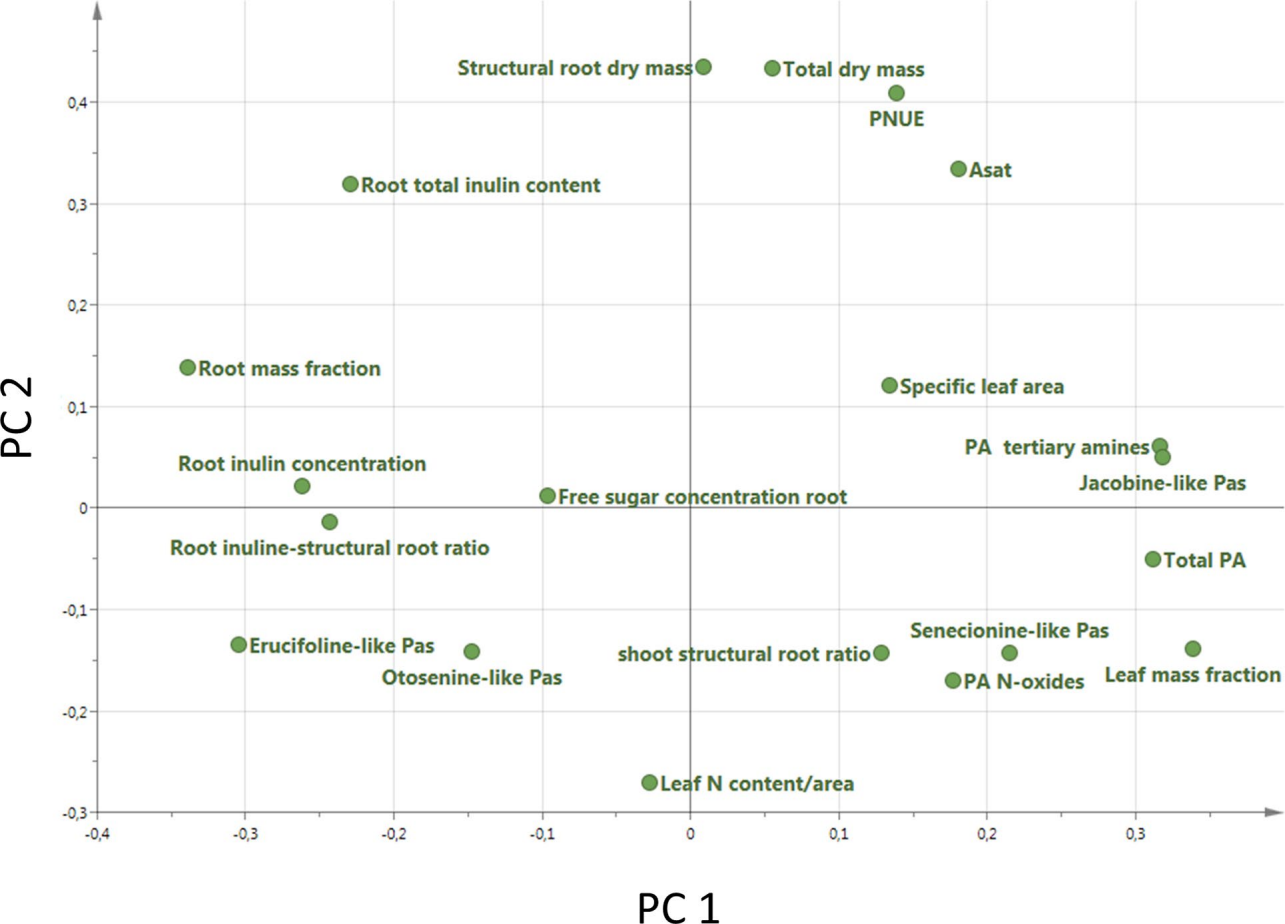
Loading plot of all 20 traits measured in this study (**Table 1**). For each trait the average value of each population was used (n = 10 Australia, n = 2 for New Zealand, n = 13 for Western North America, n = 6 for Eastern North America and n = 46 for Europe).

A PCA analysis on all 20 traits related to growth, regrowth, and defense shows that the invasive populations from different regions cluster together while they differ from the native populations (**Figure 5**). The separation of native and invasive populations in the traits related to growth, regrowth, and defense only occurs along PC1 (**Figure 5**). PC2 is not contributing to the differences between native and invasive populations. The loading plot shows that all traits contribute to the separation between native and invasive genotypes, except leaf N content, structural root dry mass, total dry mass, and free sugar concentration (**Figure 6**). The traits contributing most to the separation between invasive and native genotypes are traits related to inulin content, PA concentration, the PA groups, and to the mass fraction of leaves and roots.

We first correlated the PC1 and PC2 scores of the climate and soil with the average trait values of each population (**Table S4**), but neither PC1 nor PC2 was significantly correlated with the traits for Holm–Bonferroni corrected *p* values. The PC1 scores of the PCA plot of traits related to growth, regrowth, and defense were significantly correlated with the PC1 scores of the climate and soil PCA (**Figure S2**), explaining 6.6% of the variation, while PC2 scores of climate and soil were not significantly correlated. In the first plot, populations of the native range are mostly below the regression line while populations from the native area were mostly above the line. The residuals differed strongly and significantly between native and invasive populations (ANOVA, *F*_4,72_ = 56.94, *p* < 0.001, **Figure S3**), showing that most of the variation in PC1 of the traits is explained by the difference between native and invasive ranges. All invasive ranges, except New Zealand, differed significantly from the native range for the corrected PC score of the traits (**Figure S3**).

## Discussion

The major prediction of the EICA and SDH hypothesis is that the absence of herbivory by specialist herbivores leads to an evolutionary shift in allocation from defense to growth. In this study, we found strong evidence that invasive *J. vulgaris* genotypes grow faster compared to the native genotypes and that the increased growth is due to a higher SLA, *A*_sat_, and PNUE (**Table 1**). Furthermore, invasive genotypes had a higher LMF than native genotypes, which is positively correlated with plant growth ability (Gedroc et al., 1996; Andrews et al., 1999). At the time of harvest, invasive genotypes indeed had a larger total dry mass than the native genotypes. As *J. vulgaris* is a monocarpic plant, a higher reproductive mass directly translates into an increased fitness (Prins et al., 1990). An earlier study reported that invasive genotypes reached a 35% higher reproductive biomass compared to native ragwort plants (Joshi and Vrieling, 2005). As expected, invasive *J. vulgaris* had a higher photosynthetic capacity (*A*_sat_). Although *A*_sat_ of invasive *J. vulgaris* was higher compared to native genotypes, photosynthetic rates under climate room growth conditions do not necessarily translate into growth differences in a situation with limiting irradiance (Poorter and Oberbauer, 1993). However, photosynthetic capacity can be more fully utilized under high irradiance daylight conditions (Pons and Westbeek, 2004). The leaf nitrogen content did not differ between native and invasive genotypes; thus, the higher *A*_sat_ of invasive genotypes can be expected to contribute to a higher growth rate, and hence, the invasive genotypes have a higher PNUE (**Figure 2** and **Table 1**). This indicates that invasive genotypes may allocate a larger portion of their leaf nitrogen to increase photosynthetic capacity, while a smaller part is dedicated to other functions such as leaf cell wall proteins (Poorter and Evans, 1998; Schieving and Poorter, 1999; Pons and Westbeek, 2004). For example, Feng et al. (2009) found that invasive *Ageratina adenophora* had evolved an increased nitrogen allocation to photosynthesis and, at the same time, a reduced allocation of nitrogen to cell walls, suggesting a trade-off. Feng et al. (2009) suggested that a reduced allocation of nitrogen to cell walls leads to less resistant plants. For *J. vulgaris*, we found that invasive genotypes indeed had a lower amount of cell wall proteins per unit leaf area than native genotypes, and the specialist cinnabar moth (*T. jacobaeae*) preferred invasive genotypes over native genotypes (Lin et al., 2015a). Finally, the higher shoot to structural root ratio in the invasive *J. vulgaris* genotypes represents a redistribution of resources from root storage to growth of aboveground parts, and thus contributes to a better growth performance (**Figure 2E**).

Invasive *J. vulgaris* genotypes from the four invasive regions had, on average, a 34% lower inulin content in the roots than native genotypes, but they did not differ from each other in structural root mass (**Figure 2** and **Table 1**). This indicates that the difference in root mass between invasive and native *J. vulgaris* was solely due to the differences in root carbohydrate storage of which invasive genotypes had allocated less to the roots. Since root carbohydrate storage has been positively associated with plant regrowth after defoliation (Donaghy and Fulkerson, 1997; Aranjuelo et al., 2015), invasive *J. vulgaris* is supposed to have poorer regrowth ability. This is consistent with the findings that native *J. vulgaris* genotypes showed better regrowth after complete defoliation by clipping in a competition-free condition in a common garden experiment (Joshi and Vrieling, 2005; Lin et al., 2018) and after herbivory by the generalist *Mamestra brassicae* or the specialist *T. jacobaeae* under intraspecific competition (Lin et al., 2015b). We showed that the reduced allocation to regrowth ability in invasive *J. vulgaris* is most likely due to the absence of the selection pressure by the specialist *T. jacobaeae* in the invasive range (Lin et al., 2018).

Invasive *J. vulgaris* genotypes had, on average, higher total PA and PA tertiary amine concentrations than native genotypes. Considering the Jb chemotype plants only, the invasive genotypes had a significantly higher total tertiary amine concentration than the native genotypes. Since tertiary amines have been found to be more deterrent to insects than the PA N-oxides (Macel et al., 2005; Nuringtyas et al., 2014), the PA compositions of invasive genotypes are potentially more toxic to generalist herbivores than that of native genotypes. It has also been found that the specialist herbivore *T. jacobaeae* is attracted by tertiary amine Jb-like PAs (Macel and Vrieling, 2003; Cheng et al., 2013). The absence of *T. jacobaeae* might therefore also have helped to select for a higher tertiary amine content in the invasive range. Furthermore, our findings are in line with those of Joshi and Vrieling (2005) who also reported the absence of *J. vulgaris* Er chemotypes in the invasive range. They suggested that the Er chemotype has either not been introduced to the invasive range or has been selected against after introduction. Jb chemotype plants from the native populations contained, on average, 7.7% Er-like PAs, while in the introduced range, only trace amounts of erucifoline were present in this chemotype (**Figure 2**), suggesting that erucifoline has been selected against. Several studies have shown that generalist insect herbivores respond differently to the same PA and that the relative effects of individual PAs can differ between herbivore species (Macel et al., 2005; Cheng et al., 2013; Wei et al., 2015; Cogni and Trigo, 2016). Thus, generalist herbivores, in turn, might play an important role in the evolution and maintenance of the diversity of PAs (Macel et al., 2005). We argue that the changes in the herbivore guild towards generalist herbivores dominant in the introduced areas could have led to selection on the PA composition in invasive *J. vulgaris*. The difference in PA composition between native and invasive *J. vulgaris* genotypes might also explain our previous finding that the outcome of the competition between native and invasive genotypes was strongly dependent on whether a specialist or a generalist herbivore species was attacking (Lin et al., 2015b).

In this study, we found that all the traits of *J. vulgaris* genotypes that differed significantly among the five regions (Australia, New Zealand, Western North America and Eastern North America, and Europe) (**Table 1**) changed in the same direction in all four invasive regions (**Figure 2**). Only for the genotypes from New Zealand some traits showed no significant difference compared to the native genotypes. It should be remarked that the sample size for New Zealand is low, as only two populations were used. Nevertheless, all traits deviated in the predicted direction, even when not significant. The result of this study is consistent with our previous studies, which showed that regrowth, structural defenses, root–shoot ratio, and competitive ability all changed in different invasive regions in the same direction (Lin et al., 2015a; Lin et al., 2015b; Lin et al., 2018). Here, we also show that *A*_sat_, PNUE, chemical defense, and inulin allocation to roots of invasive *J. vulgaris* genotypes are showing parallel changes in all invasive regions.

The PCA showed that all measured traits contributed to the separation between native and invasive genotypes along PC1 (**Figure 5**). The loading plot shows that the traits contributing mostly to the separation along PC1 were defense, storage, and allocation-related traits (**Figure 6**). Traits related to photosynthetic capacity (SLA, *A*_sat_, PNUE) contributed less. Apparently, a suite of traits changes simultaneously in *J. vulgaris* when released from specialist herbivores.

The invasive populations from each region were separated in the PCA based on combined climatic and soil variables but not in the PCA based on plant traits (**Figures 4** and **5**). PC1 of the traits that separates native and invasive populations is correlated with PC1 of the climate and soil PCA. However, the explained variance is small (6.6%, **Figure S2**). This shows that the soil and climatic variables only explain the changes in traits between native and invasive genotypes for a very small part. Correcting the PC scores of the trait values for the influence of soil and climate shows that large differences between the native and invasive range remain (**Figure S3**). It suggests that the herbivore guild plays an important role: all invasive ranges have a higher corrected PC1 score than the native range. Only the corrected values for New Zealand do not significantly differ from the native range. Thus, soil and climatic factors can be largely ruled out as important selective forces for the traits studied here. Although we can largely rule out soil and climatic conditions, still other yet unknown factors may play a role. For instance, Williams et al. (2010) found that disturbances of the soil explained differences in population growth between native and invasive ranges of *Cynoglossum officinale*. In addition, it should be noted that soil data extracted from the ISRIC database might not have the appropriate resolution to provide the necessary information how these variables act on plant performance at the local scale as there might be considerable spatial variation in these variables even within few meters.

In conclusion, we found invasive *J. vulgaris* genotypes to have a higher growth, enabled by an increase in photosynthetic rates, LMA, and to a lesser extent SLA. In addition, the shoot to structural ratio in invasive plants is larger, and less resources are allocated to inulin storage in the roots. Both traits will contribute to an increased growth rate as well. Invasive *J vulgaris* genotypes increased their qualitative defense, through an increased production of PA tertiary amines, which possess higher toxicity. These results fully supported our hypotheses and the SDH hypothesis. They only partly support the EICA hypothesis, as the PAs defenses against generalists are increased in the invasive area, which is not predicted by the EICA. The results show that a logically connected suite of traits related to growth, regrowth, and defense is prone to change when the selective pressure changes due to the absence of specialist herbivores after invasion.

## Author Contributions

TL, PK, and KV conceived the ideas and designed methodology; TL, TP, and PM collected the data; TL, PK, and KV analyzed the data; TL led the writing of the manuscript. All authors contributed critically to the drafts and gave final approval for publication.

## Funding

TL thanks the Education Department of Sichuan Province (Grant No. 18ZA0389) and National students’ platform for innovation and entrepreneurship training program (Grant No. 201810626067) for financial support.

## Conflict of Interest Statement

The authors declare that the research was conducted in the absence of any commercial or financial relationships that could be construed as a potential conflict of interest.
