# Supplementary material for: Evolution of Increased Photosynthetic Capacity and Its Underlying Traits in Invasive Jacobaea vulgaris
Source: Front Plant Sci. 2019 Aug 8;10:1016. doi: 10.3389/fpls.2019.01016 (PMC6694182; doi:10.3389/fpls.2019.01016)
Supplement: Supplementary file 1 [file DataSheet_1.docx]

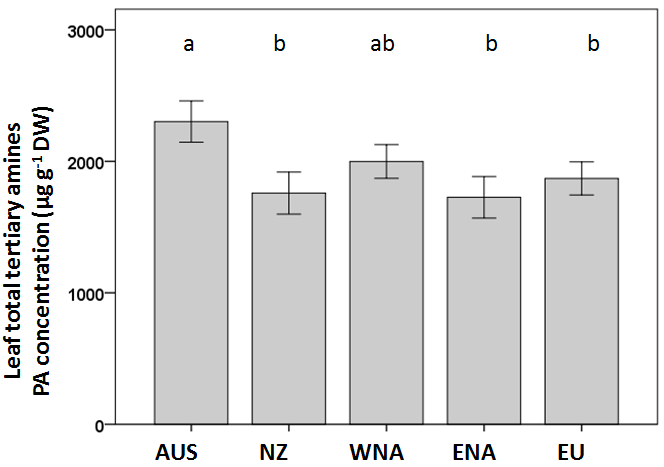

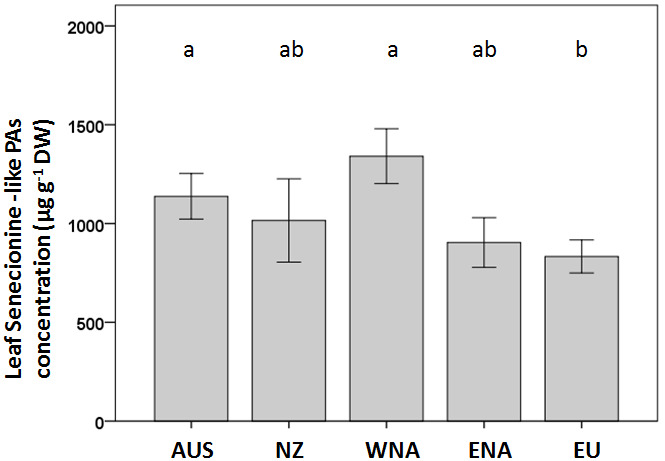


(b)

(a)


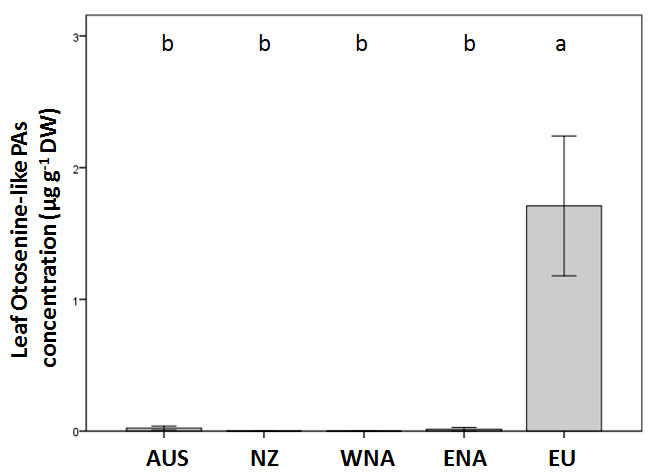

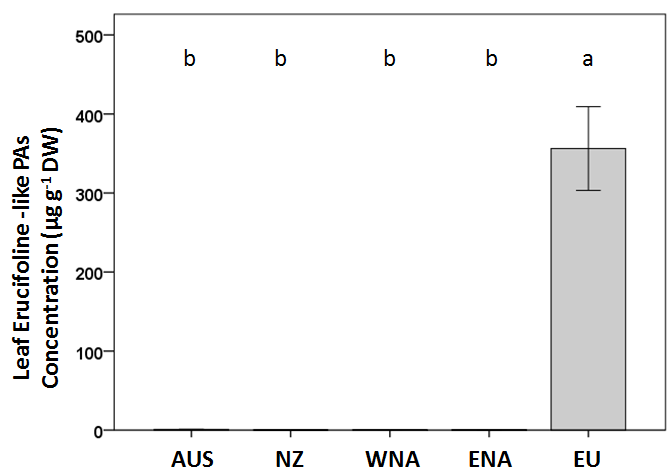


(d)

(c)

Fig. S1. Mean value of leaf pyrrolizidine alkaloid concentration of Jb chemotypes of *Jacobaea vulgaris* populations from four invasive regions (Aus= Australia, NZ=New Zealand, WNA = Western North America and ENA= Eastern North America) and the native region (EU= Europe).(a) Total tertiary amines PAs, (b) Senecionine-like PAs, (c) Erucifoline-like PAs, (d) Otosenine-like PAs. Values are means ± SE. Different letters indicate significant differences among regions at p <0.05 according to a post hoc LSD test (For the nested ANOVA results see Table 2).


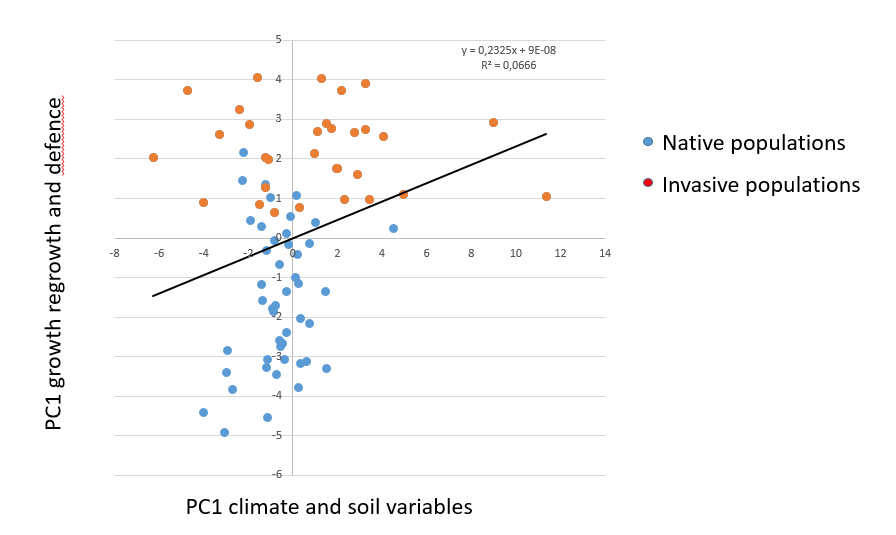


Fig. S2 Correlations between PC1 of climate and soil variables (Fig.3) and PC1 of growth, regrowth and defense variables (Fig. 4).


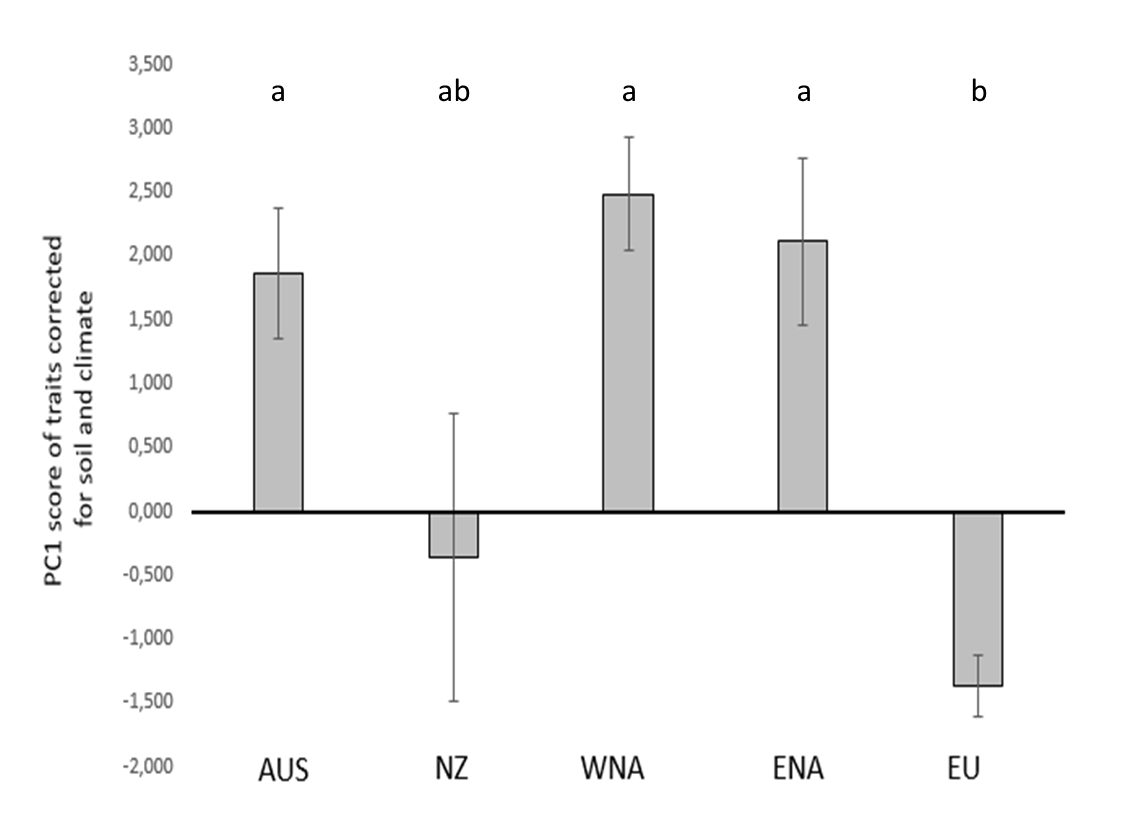


Fig. S3. PC1 scores of trait values corrected for soil and climate for all regions (see Fig. S2). Different letters indicate significant differences with a post hoc test after ANOVA (F_4,72_ = 22.26, p<0.001)
