# Supplementary material for: Evolution of Increased Photosynthetic Capacity and Its Underlying Traits in Invasive Jacobaea vulgaris
Source: Front Plant Sci. 2019 Aug 8;10:1016. doi: 10.3389/fpls.2019.01016 (PMC6694182; doi:10.3389/fpls.2019.01016)
Supplement: Supplementary file 2 [file DataSheet_2.docx]

Table S1. Origin of the seeds of 46 native and 31 invasive populations of

*J. vulgaris* used in this study.

| *Origin* | *Country/Region* | *Location* | *Longitude* | *Latitude* |
| --- | --- | --- | --- | --- |
| Invasive | Western NorthAmerica | Cooper Mountain, Oregon | 122°53' W | 45°27' N |
|  |  | Indian Creek, Oregon | 117°49' W | 44°01' N |
|  |  | Sunrise Hill, Montana | 115°00' W | 48°15'N |
|  |  | West Crestmont, Oregon | 121°51' W | 45°22' N |
|  |  | Island Lake, Oregon | 122°37' W | 45°25' N |
|  |  | Larch Slope, Oregon | 122°10' W | 45°30' N |
|  |  | Conrad Spur, Montana | 111°58' W | 48°09' N |
|  |  | No Bear Road, Oregon | 120°33' W | 43°48' N |
|  |  | Island Lake Road, Montana | 114°59' W | 48°12'N |
|  |  | Little Wolf, Montana | 115°00' W | 48°21' N |
|  |  | Kootenai National Forest, Montana | 114°53' W | 48°17' N |
|  |  | Salem, Oregon | 122°95' W | 44°87' N |
|  |  | Cochran Creek, Oregon | 122°58' W | 44°25' N |
|  | EasternNorthAmerica | Cardigan, Prince Edward Island | 63°37' W | 46°13' N |
|  |  | Charlottetown, Prince Edward Island | 63°07' W | 46°14' N |
|  |  | Green Cables, Prince Edward Island | 63°22' W | 46°29' N |
|  |  | Clinton, Prince Edward Island | 63°32' W | 46°26' N |
|  |  | Cavendish Beach, Prince Edward Island | 63°24' W | 46°29' N |
|  |  | Marco Polo Campground, Prince Edward Island | 63°22' W | 46°29' N |
|  | Australia | Franklin, Tasmania | 147°01' E | 43°05' S |
|  |  | Dairy Plains 1, Tasmania | 146°31' E | 41°38' S |
|  |  | Dairy Plains 2,Tasmania | 146°32' E | 41°34' S |
|  |  | Wild Dog Road, Victoria | 143°40' E | 38°41' S |
|  |  | Beech Forest, Victoria | 143°33' E | 38°38' S |
|  |  | Barramunga, Victoria | 143°41' E | 38°34' S |
|  |  | Cape Schanck, Victoria | 144°54' E | 38°27' S |
|  |  | Turton's Creek, Victoria | 146°15' E | 38°33' S |
|  |  | Targa, Tasmania | 147°23' E | 41°18' S |
|  |  | Franklin, Tasmania | 147°01' E | 43°04' S |
|  | New Zealand | Fox Glacier, South island | 170°01' E | 43°27' S |
|  |  | Tongariro National Park,North island | 175°34' E | 39°14' S |
| Native | Belgium | Bertogne1 | 05°40' E | 50°05' N |
|  |  | Bertogne2 | 05°40' E | 50°05' N |
|  |  | Spa | 05°50' E | 50°29' N |
|  |  | Brussels | 04°25' E | 50°51' N |
|  |  | Louvain-la-Neuve | 04°37' E | 50°40' N |
|  | Denmark | Sundstrup | 09°18' E | 56°36' N |
|  |  | Hovborg | 08°56' E | 55°36' N |
|  |  | Engesvang | 09°21' E | 56°10' N |
|  | Finland | Masala, Kirkkonummi | 24°31' E | 60°09' N |
|  |  | Inkoo, Kirkkonummi | 24°00' E | 60°02' N |
|  | France | Mont Saint Michel, Normandie | 01°32' W | 48°37' N |
|  |  | Rouen, Normandie | 01°05' W | 49°26' N |
|  |  | Pontorson, Normandie | 01°36' W | 48°33' N |
|  |  | Les Essarts, Pays-de-la-Loire | 01°10' W | 46°07' N |
|  |  | Lesparre-Medoc, Aquitaine | 00°54' W | 45°17' N |
|  |  | Brillac, Bretagne | 02°48' W | 47°32' N |
|  |  | Lamotte-Beuvron | 02°01' W | 47°36' N |
|  |  | Lourdes, Midi-Pyrénées | 00°13' W | 43°09' N |
|  | Germany | Halle, Sachsen | 11°58' E | 51°29' N |
|  |  | Lubeck, Schleswig-Holstein | 10°42' E | 54°05' N |
|  |  | Nassenheide, Brandenburg | 13°14' E | 49°14' N |
|  |  | Pfingstberg, Brandenburg | 13°52' E | 53°08' N |
|  | Hungary | Csokvaomány | 20°22' E | 48°10' N |
|  |  | Lénárddaróc | 20°22' E | 48°08' N |
|  | Netherlands | Meijendel | 04°20' E | 52°07' N |
|  |  | Wageningen | 05°34' E | 52°10' N |
|  |  | Mossel | 05°45' E | 52°03' N |
|  |  | Gees | 06°41' E | 52°44' N |
|  |  | Texel | 04°48' E | 53°05' N |
|  | Norway | Malvik | 10°37' E | 63°25' N |
|  | Poland | Pulawy | 21°59' E | 51°24' N |
|  | Spain | Covadonga | 04°45' W | 43°04' N |
|  | Sweden | Lund | 13°13' E | 55°43' N |
|  |  | St Olofsholm, Gotland | 18°54' E | 57°43' N |
|  | Switzerland | Mettembert | 07°19' E | 47°23' N |
|  |  | Saint-Imier | 07°00' E | 47°09' N |
|  | UK | Falls of Leny, Scotland | 04°16' W | 56°15' N |
|  |  | Bentley, Hampshire | 00°51' W | 51°10' N |
|  |  | Alice Holt Forest, Hampshire | 00°50' W | 51°10' N |
|  |  | Silwood Park, Berkshire | 00°38' W | 51°24' N |
|  |  | Saint Andrews, Scotland | 02°47' W | 56°20' N |
|  |  | Sevenoaks Weald, Kent | 00°12' W | 51°14' N |
|  |  | Marsh Green, Devon | 03°21' W | 50°44' N |
|  |  | Threemilestone, Cornwall | 05°05' W | 50°16' N |
|  |  | Enfield, Greater London | 00°03' W | 51°40' N |
|  |  | Corston, Bath | 02°26' W | 51°23' N |

Table S2. The average values of 19 available bioclimatic variables of Europe, Australia, New Zealand, Western North America and Eastern North America. Values are mean values ± SE.

| *Bioclimatic variables* | *Europe* | *Australia* | *New Zealand* | *Western North America* | *Eastern North America* |
| --- | --- | --- | --- | --- | --- |
| Annual Mean Temperature (°C) | 8.87±0.28 | 11.57±0.39 | 8.58±1.21 | 8.02±0.82 | 5.61±0.07 |
| Mean Monthly Temperature Range (°C) | 7.60±0.17 | 8.99±0.25 | 9.39±0.41 | 12.59±0.61 | 8.42±0.06 |
| Isothermality | 32.90±0.70 | 48.98±0.49 | 45.67±0.10 | 40.75±1.01 | 24.29±0.17 |
| Temperature Seasonality (°C) | 593.0±16.1 | 334.6±5.7 | 398.0±16.4 | 672.2±45.8 | 945.3±4.3 |
| Max Temperature of Warmest Month (°C) | 21.48±0.28 | 21.79±0.26 | 19.60±1.40 | 26.17±0.71 | 23.38±0.10 |
| Min Temperature of Coldest Month (°C) | -1.86±0.78 | 3.43±0.55 | -0.95±0.45 | -5.12±1.64 | -11.33±0.13 |
| Temperature Annual Range (°C) | 23.34±0.51 | 18.36±0.44 | 20.55±0.95 | 31.28±1.91 | 34.72±0.04 |
| Mean Temperature of Wettest Quarter (°C) | 9.37±0.75 | 7.68±0.44 | 7.15±4.48 | 2.12±1.61 | -1.78±0.94 |
| Mean Temperature of Driest Quarter (°C) | 6.64±0.86 | 15.75±0.38 | 8.24±3.81 | 14.45±1.77 | 11.29±2.76 |
| Mean Temperature of Warmest Quarter (°C) | 16.17±0.22 | 15.75±0.38 | 13.38±1.33 | 16.49±0.59 | 17.08±0.03 |
| Mean Temperature of Coldest Quarter (°C) | 1.72±0.43 | 7.45±0.43 | 3.56±0.88 | 0.08±1.31 | -5.93±0.14 |
| Annual Precipitation (mm) | 804.2±32.5 | 1149.4±78.7 | 3642±584 | 1089±208 | 1108.5±4.2 |
| Precipitation of Wettest Month (mm) | 89.1±3.8 | 140.3±11.6 | 372±49 | 170.2±35.8 | 117.2±1.2 |
| Precipitation of Driest Month (mm) | 47.54±2.19 | 52±2.25 | 212±52 | 22.31±2.69 | 80.5±0.5 |
| Precipitation Seasonality (mm) | 21.53±1.17 | 29.80±1.99 | 17.27±2.19 | 50.85±5.08 | 14.47±0.54 |
| Precipitation of Wettest Quarter (mm) | 252.2±10.5 | 388.2±31.4 | 1065±128 | 484±103 | 336.7±2.7 |
| Precipitation of Driest Quarter (mm) | 155.1±7.0 | 175.7±8.5 | 743±148 | 97.8±11.1 | 246.7±1.4 |
| Precipitation of Warmest Quarter (mm) | 204.5±7.3 | 175.7±8.5 | 823±228 | 114.7±10.3 | 252.2±0.7 |
| Precipitation of Coldest Quarter (mm) | 204±12.2 | 384.7±32.8 | 914±23 | 441.5±99.6 | 304.7±2.0 |

Mean Monthly Temperature Range=Mean of monthly (max temp - min temp); Isothermality= Mean Monthly Temperature Range/ Temperature Annual Range; Temperature Annual Range= Max Temperature of Warmest Month- Min Temperature of Coldest Month.

Table S3. The average values of 10 available bioclimatic variables of Europe, Australia, New Zealand, Western North America and Eastern North America. Values are mean values ± SE.

| *Soil variables* | *Europe* | *Australia* | *New Zealand* | *Western North America* | *Eastern North America* |
| --- | --- | --- | --- | --- | --- |
| Bulk density in t / cubic-meter | 1.22±0.02 | 1.05±0.02 | 0.85±0.05 | 1.12±0.05 | 0.98±0.03 |
| Soil texture fraction clay in percent | 17.07±0.94 | 22.10±1.07 | 20.05±3.50 | 17.54±1.88 | 13.33±1.09 |
| Coarse fragments in volumetric percent | 10.50±0.66 | 13.00±0.49 | 11.00±3.00 | 8.77±1.76 | 4.50±0.56 |
| Soil texture fraction silt in percentage | 35.30±1.75 | 19.50±1.25 | 31.50±3.50 | 44.31±2.05 | 30.50±1.26 |
| Soil texture fraction sand in percentage | 47.72±2.37 | 58.00±2.09 | 47.00±0.00 | 38.15±2.91 | 56.17±2.12 |
| Cation exchange capacity (fine earth fraction) in cmolc/kg | 28.67±1.06 | 34.00±2.71 | 35.50±6.50 | 31.92±1.85 | 24.33±1.84 |
| Soil organic carbon content (fine earth fraction) in permilles | 119.74±8.28 | 66.60±7.73 | 119.00±10.00 | 123.38±21.23 | 137.50±5.05 |
| Soil pH in H2O | 5.93±0.08 | 5.70±0.11 | 5.25±0.15 | 6.05±0.20 | 4.98±0.12 |
| Soil pH in KCl | 4.97±0.208 | 5.15±0.11 | 4.40±0.30 | 5.09±0.23 | 4.48±0.12 |
| Volumetric water content at wilting point pF 4.2 | 24.17±0.66 | 23.90±0.90 | 29.50±1.50 | 27.46±0.95 | 23.67±0.92 |

Table S4. P values of Pearson correlation coefficients between all measured trait values and PC1 and PC2 scores of the PCA of all climate and soil variables. A star indicates significant p values before Holm-Bonferroni correction for multiple testing. After correcting for multiple testing per PC score all significant correlations became not significant. N = 77.

| Trait | pc 1 | pc 2 |
| --- | --- | --- |
| Total dry mass | 0,060 | -0,139 |
| Leaf mass fraction | ,246^*^ | 0,175 |
| Root mass fraction | -,246^*^ | -0,175 |
| Specific leaf area | 0,120 | -0,078 |
| Structural root dry mass | -0,038 | -0,177 |
| shoot structural root ratio | ,238^*^ | 0,151 |
| Leaf N content/area | -0,039 | 0,193 |
| Asat | 0,143 | 0,037 |
| PNUE | 0,125 | -0,082 |
| Total PA | 0,127 | 0,083 |
| PA tertiary amines | ,231^*^ | 0,016 |
| PA N-oxides | -0,055 | 0,133 |
| Senecionine-like Pas | 0,075 | 0,185 |
| Jacobine-like Pas | 0,193 | 0,026 |
| Erucifoline-like Pas | -,301^**^ | -0,058 |
| Otosenine-like Pas | -0,224 | 0,057 |
| Root inulin concentration | -0,036 | -0,053 |
| Root total inulin content | -0,107 | -0,206 |
| Free sugar concentration root | -0,128 | -0,104 |
| Root inuline-structural root ratio | -0,041 | -0,062 |
